# Identification of Pathogenic Mutations and Investigation of the NOTCH Pathway Activation in Kartagener Syndrome

**DOI:** 10.3389/fgene.2019.00749

**Published:** 2019-08-22

**Authors:** Yongjian Yue, Qijun Huang, Peng Zhu, Pan Zhao, Xinjuan Tan, Shengguo Liu, Shulin Li, Xuemei Han, Linling Cheng, Bo Li, Yingyun Fu

**Affiliations:** ^1^Key Laboratory of Shenzhen Respiratory Diseases, Department of Pulmonary and Critical Care Medicine, Shenzhen Institute of Respiratory Disease, The First Affiliated Hospital of Southern University of Science and Technology, The Second Clinical Medical College of Jinan University, Shenzhen People’s Hospital, Shenzhen, China; ^2^Central Lab of Shenzhen Pingshan People’s Hospital, Shenzhen, China; ^3^Clinical Medical Research Center, The First Affiliated Hospital of Southern University of Science and Technology, The Second Clinical Medical College of Jinan University, Shenzhen People’s Hospital, Shenzhen, China; ^4^State Key Laboratory of Respiration Diseases, The First Affiliated Hospital of Guangzhou Medical University, Guangzhou, China; ^5^Department of Pediatric, First Affiliated Hospital of Southern University of Science and Technology, The Second Clinical Medical College of Jinan University, Shenzhen People’s Hospital, Shenzhen, China

**Keywords:** Kartagener syndrome, compound heterozygous, *CCDC40*, *DNAH1*, *DNAH5*, *DNAI1*, NOTCH pathway

## Abstract

Primary ciliary dyskinesia (PCD), a rare genetic disorder, is mostly caused by defects in more than 40 known cilia structure-related genes. However, in approximately 20–35% of patients, it is caused by unknown genetic factors, and the inherited pathogenic factors are difficult to confirm. Kartagener syndrome (KTS) is a subtype of PCD associated with situs inversus, presenting more complex genetic heterogeneity. The aim of this study was to identify pathogenic mutations of candidate genes in Chinese patients with KTS and investigate the activation of the heterotaxy-related NOTCH pathway. Whole-exome sequencing was conducted in five patients with KTS. Pathogenic variants were identified using bioinformatics analysis. Candidate variants were validated by Sanger sequencing. The expression of the NOTCH pathway target genes was detected in patients with KTS. We identified 10 KTS-associated variants in six causative genes, namely, *CCDC40*, *DNAH1*, *DNAH5*, *DNAH11*, *DNAI1*, and *LRRC6*. Only one homozygote mutation was identified in *LRRC6* (c.64dupT). Compound heterozygous mutations were found in *DNAH1* and *DNAH5*. Six novel mutations were identified in four genes. Further analyses showed that the NOTCH pathway might be activated in patients with KTS. Overall, our study showed that compound heterozygous mutations widely exist in Chinese KTS patients. Our results demonstrated that the activation of the NOTCH pathway might play a role in the situs inversus pathogenicity of KTS. These findings highlight that Kartagener syndrome might be a complex genetic heterogeneous disorder mediated by heterozygous mutations in multiple PCD- or cilia-related genes.

## Introduction

Primary ciliary dyskinesia (PCD) is a sinopulmonary disease caused by dysfunctional immotile or dyskinetic respiratory cilia. It is an autosomal recessive disorder and clinically characterized by respiratory distress, tympanitis, sinusitis, bronchiectasis, and male infertility in approximately 50% of patients. Male infertility usually caused by aberrant sperm flagella function results from immotile or dyskinetic cilia. Further, PCD affects approximately 1 in 15,000 individuals of the general population (Bush et al., 2007). It was reported that patients with heterotaxy and airway ciliary dysfunction were enriched for mutations in the PCD genes, but interestingly, these were all heterozygous (Wilson et al., 2009). Kartagener syndrome (KTS;MIM# 244400), a subtype of PCD presenting with the phenotype situs inversus, might have a more complex genetic heterogeneity.

The nasal nitric oxide measurement and brush biopsy are typical diagnostic methods for PCD. The diagnosis of PCD is challenging due to phenotypic heterogeneity. Moreover, owing to the non-specificity of symptoms and lack of an early diagnostic method, the diagnosis of PCD is usually missed or delayed (Hosie et al., 2014; Lucas et al., 2014). Because of the lack of a reference standard for PCD diagnosis, a combination of tests is required (Lucas et al., 2016). Definitive diagnosis is usually confirmed through transmission electron microscopy (TEM) to visualize the ultrastructure of cilia, but approximately 30% of patients show no defect in TEM. Furthermore, there is a lack of evidence-based medicine in the management of PCD (Lucas et al., 2014). Therefore, the early diagnosis of PCD is important to select an appropriate management to prevent irreversible damage. With the rapid advances in next-generation sequencing, genetic-based studies have increased our understanding of the pathogenesis of even PCD with remarkable genetic heterogeneity. Diagnostic gene panel has been used for several diseases, and a genetic testing panel for PCD diagnosis is being developed (Paff et al., 2018; Takeuchi et al., 2018). As an autosomal recessive disorder, the inherited mutations of ciliary function-related genes are the main genetic risk factors of PCD. Most of the genetic mutations in PCD contribute to defective cilia structure. Some patients with PCD due to mutations also showed normal motility and ultrastructure, but reduced cilia numbers (Wallmeier et al., 2014). Mutations of ciliary function-related genes can cause immotile cilia due to congenital ultrastructural abnormalities. The prevalence of PCD has a relatively higher rate in consanguineous families (Onoufriadis et al., 2014). To date, genetic mutations in over 40 genes, including *DNAH5*, *CCD39*, and *DYX1C1*, account for an estimated 65% of individuals (Olbrich et al., 2002; Merveille et al., 2011; Tarkar et al., 2013). *DNAH5* mutations cause approximately 50% of outer dynein arm (ODA) defects, whereas SPAG1 mutations cause approximately 10% of inner dynein arm (IDA) and ODA defects (Hornef et al., 2006; Knowles et al., 2013; Lucas et al., 2014).

The pathogenic genes reported previously have improved genetic-based diagnosis. Based on the pathogenic mutations of known genes involved in PCD, genetic testing presents a possibility to identify high-risk populations among familial siblings with PCD. However, the genetic factors in approximately 20–35% of patients with PCD are unknown, and the pathogenicity of novel mutations in known PCD genesis is also difficult to confirm based on a functional study. The genetic defect has not yet been well elucidated in some patients. Previously, one study demonstrated that *GALNT11* is a heterotaxy disease candidate gene (Fakhro et al., 2011). Further, another study showed that GALNT11 O-glycosylation modified NOTCH, which balances motile and immotile cilia in left–right patterning and affects the etiology of heterotaxy (Boskovski et al., 2013). However, whether the NOTCH pathway is associated with KTS remains unclear. In the present study, we aimed to identify pathogenic mutations and investigate the NOTCH pathway activation in Chinese patients with KTS. Our study would expand our knowledge on the spectrum of PCD- or cilia-related genes, thereby contributing to the genetic diagnosis and counseling of KTS patients.

### Materials and Methods

#### Subjects

Five patients with KTS and nine familial control subjects were recruited for the present study conducted in the Second Clinical Medical College of Shenzhen People’s Hospital and Institute of Guangzhou Respiratory Diseases (Guangdong, China) from December 2013 to July 2019. The control subjects included in the study were the parents of KT4 and LEI, father of KT2, mother of KT5, and the father, sister, brother, and uncle of KT1 (**Table 1**). Patients with KTS were diagnosed based on the presence of chronic sinusitis, situs inversus, bronchiectasis, and ciliary ultrastructural defects. Demographic characteristics and medical history were recorded, including age and clinical test results. Informed consent was obtained from each patient enrolled in the study. Research and ethics approval was obtained from the ethics committee of the Shenzhen People’s Hospital.

**Table 1:** Demographics of the KTS participants.

KTS ID	Age	Gender	Inflammation	Situs inversus	Bronchiectasis	Bronchitis
KT1	7	Female	Respiratory infections	Yes	Yes	Yes
KT2	8	Male	Respiratory infections	Yes	Yes	Yes
LEI	6	Male	Rhinosinusitis	Yes	Yes	Yes
KT4	7	Female	Respiratory infections	Yes	Yes	Yes
KT5	3	Male	Normal	Yes	No	No

#### Bronchoscopy and Electron Microscopy

High-resolution computed tomography and magnetic resonance imaging were performed to check for bronchiectasis or situs inversus. Nasal mucosa and bronchial cilia were obtained for electron microscopy. Transmission electron microscopic examination (Kingmed, China) was performed to identify the ultrastructural abnormality of cilia. Bronchoscopic examination was performed for PCD diagnosis.

#### Next-Generation Sequencing and Bioinformatics Analysis

High-quality genomic DNA was extracted from blood samples of patients, and exome capture was performed using the Agilent SureSelect V6 Capture Kit according to the manufacturer’s protocols (Agilent, CA, USA). An enriched library targeting the exome was subjected to massive parallel sequencing using NovaSeq platform (Illumina). The variants were called using Genome Analysis Toolkit (GATK) and annotated using ANNOVAR (Wang et al., 2010). We only focused on the genes with nonsynonymous, Indel, or deletion variants in the coding region. Later, all variants were filtered by region, frequency (MAF > 5%), and functional prediction. The following databases were used as the reference: 1,000 genome, dbSNP, and gnomAD (http://gnomad.broadinstitute.org/). 

#### Candidate Gene Selection and Pathogenicity Assessment

Previous studies have reported that more than 40 genes are related to PCD. Based on previous panel analyses and genetic studies on PCD (Guo et al., 2017; Takeuchi et al., 2018; Yang et al., 2018), 43 genes were selected as candidate genes, which were highly associated with the pathogenesis of PCD (**Supplemental Table S1**). A gene-based filtering analysis was conducted among all variants of the five patients with PCD. To assess the pathogenesis of the variants, various protein functions were predicted using bioinformatic programs, including Sift, Polyphen-2, MutationTaster, and CADD (combined annotation dependent depletion). The interpretation of variants followed the American College of Medical Genetics and Genomics (ACMG) guideline (Richards et al., 2015). An evolutionary conservation analysis of the identified variants was conducted using CLUSTALW on orthologs of different mammal species.

#### Sanger Sequencing Validation

Genomic DNA was extracted from whole blood samples using the QIAamp DNA Blood Kit (QIANGEN, Hilden, Germany) according to the manufacturer’s instruction. All primers were designed using IDT PrimerQuest Tool online software (https://sg.idtdna.com/Primerquest/Home/Index). All gene segments were amplified using the standard PCR. The candidate variants among patients with PCD and control subjects were genotyped using Sanger genotyping platform. The sequences were analyzed using Chromas software (Technelysium Pty Ltd).

#### Detection of NOTCH Signaling Pathway Activation

Blood samples of KTS and control subjects were collected into Na-citrate-containing Vacutainer tube. Total RNA was extracted from whole blood using animal tissue RNA Purification Kit (TRK-1002, LC Sciences, Houston, USA). To confirm whether the Notch pathway was abnormally activated in patients with PCD, real-time PCR was conducted for the Notch pathway–related and downstream target genes, including *ADAM17*, *Cyclin D3*, *p21*, *MYC*, and *HES1*. Primers were obtained from the database of PrimerBank (https://pga.mgh.harvard.edu/primerbank/) or designed using Integrated DNA Technologies online software (**Supplemental Table S2**).

#### Statistical Analysis

All statistical analyses were carried out using the software SPSS 19 (IBM, New York, USA). Chi square and Bonferroni (*post hoc*) tests were used for cross-table and multiple comparisons, respectively. The results with P < 0.05 were considered statistically significant.

### Results

#### Characteristics of Patients and Diagnosis of KTS

**Figure 1:**
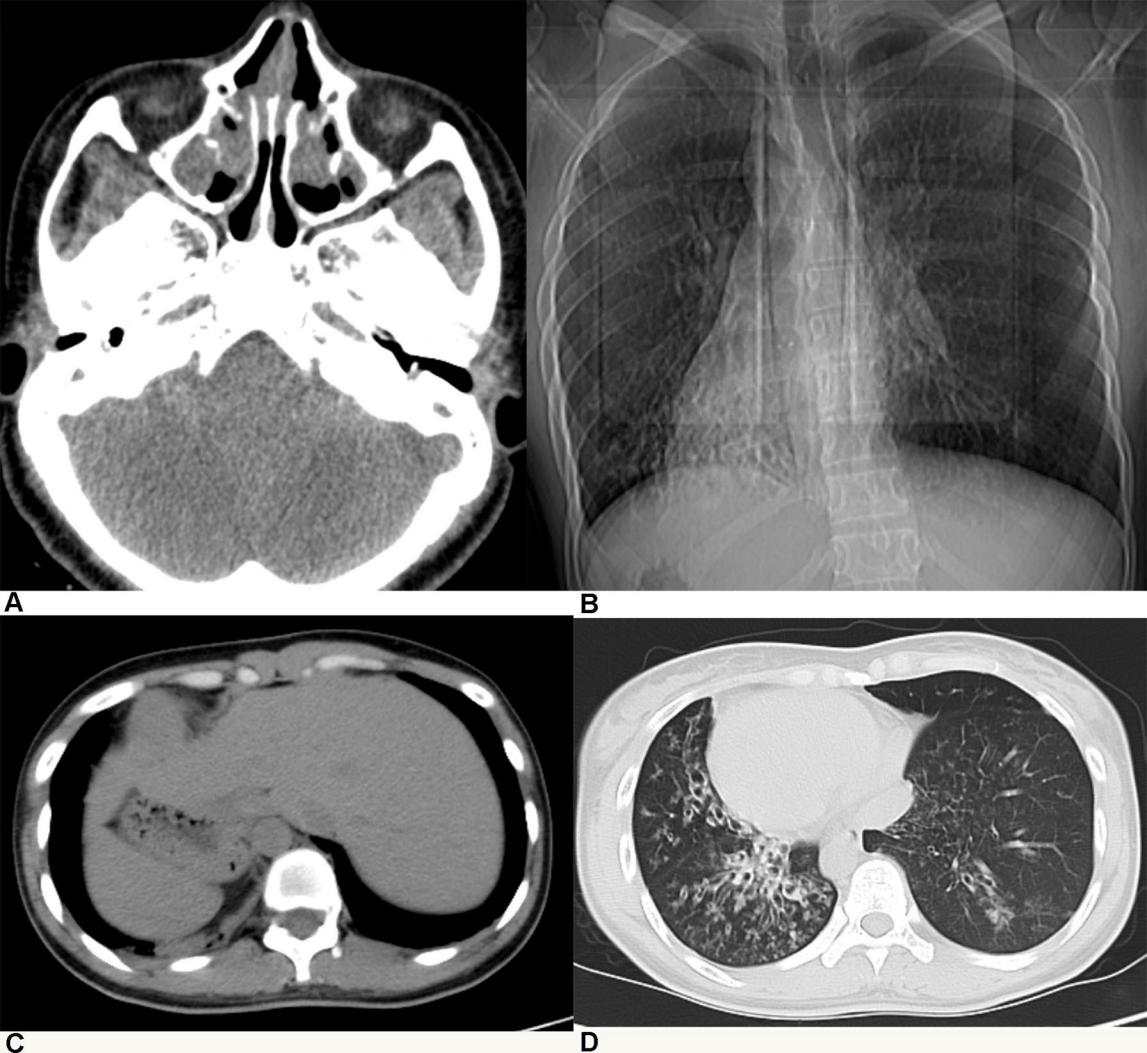
Patients with Kartagener syndrome diagnosed by radiological examination: **(A)** chronic sinusitis, **(B**, **C)** situs inversus, and **(D)** diffuse bronchiectasis.

**Figure 2:**
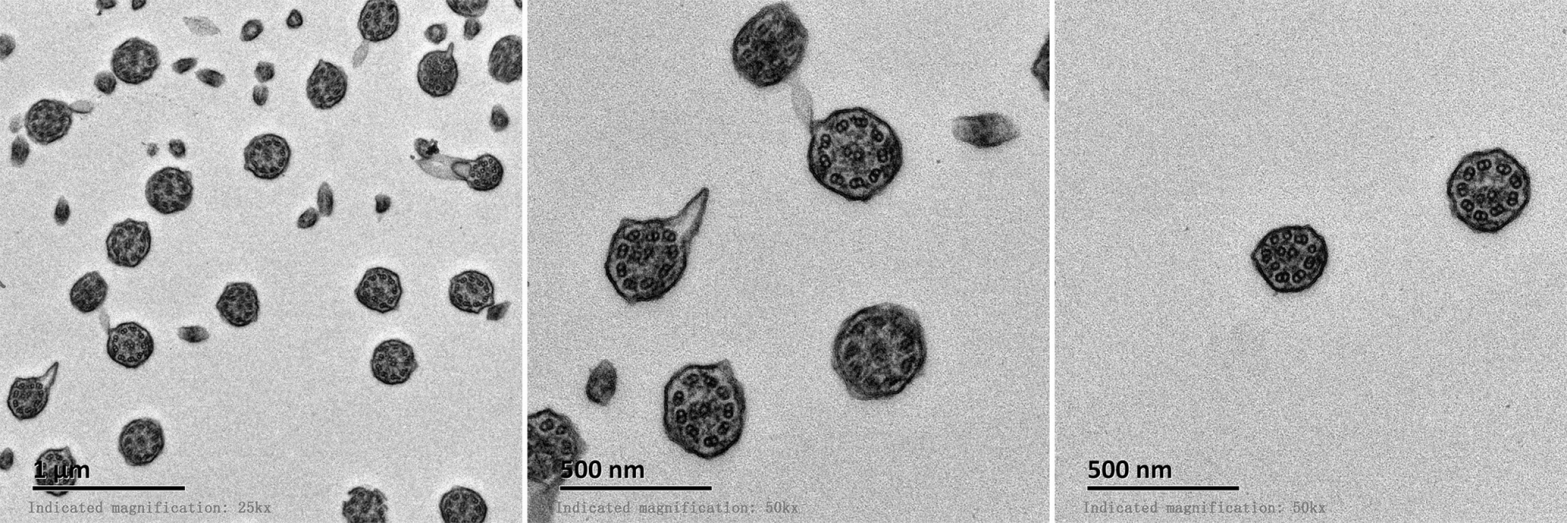
Electron microscopic examination of bronchial cilia showed abnormal 9 + 2 structure and deletion of inner or outer dynein arms.

All patients with KTS had a history of recurrent cough, expectoration, and rhinorrhea since the age of diagnosis at childhood (**Table 1**). Chest computed tomography images showed that all the patients had symptoms of KTS, including the classic phenotype of situs inversus (**Figure 1**). The family members (father) of two subjects showed slight bronchiectasis or sinusitis, but without situs inversus. These were taken as reference control subjects without KTS. Routine semen analysis and electron microscopic examination showed infertility in adult male patients and multiple ultrastructural abnormalities of cilia in both bronchial and nasal mucosa, respectively (**Figure 2**). The structural abnormalities of cilia included disarrangement, reduction, and absence of IDA and ODA structures of the subject LEI.

#### Candidate Gene Rare Variants Identified Using Filtering Analysis

To identify mutations in the Chinese population with KTS, 10 familial normal control subjects were used as annotation controls. We filtered variants to obtain rare mutations with the standard criteria as described in the methodology. With further filtering using integrated bioinformatics pipeline and software, we identified 30 variants of candidate genes (**Supplemental Table S3**). All KTS subjects carried coding mutations in *DNAH1* in the present study. Regarding the known gene variants, we changed the filtering cutoff frequency to 0.01 (gnomAD_all) to obtain all the rare coding variants in the known candidate genes of KTS. In total, 19 variants of the known candidate genes were identified (**Tables 2** and **3**). The pathogenicity of variants was assessed by four *in silico* software. All variants were interpreted based on the ACMG guideline (**Supplemental Table S3**). Nine variants were considered not associated with disease as they were benign or uncertain significance variants (VUS) based on the frequency in the Asian population and ACMG classification (**Table 2**). Six novel mutations were identified in four genes, which were not included in the dbSNP and gnomAD databases (**Table 3**). Two novel mutations of *DNAH5* were identified in the patient KT4. Only one homozygous variant was identified in *LRRC6* (c.64dupT), which is the likely cause for the genetic disorder in KT1 (**Table 3**). The two heterozygous mutations of *DNAH1* in KT1 were compound heterozygous. The variants in *DNAI1* and *DNAH1* might contribute to the phenotype of KT1. Advanced analysis showed no homozygous mutations in the known candidate genes among patients with KTS (data not shown).

**Table 2:** The identified likely benign variants of candidate genes in Kartagener syndrome (KTS).

Chr	Start	refGene	AAChange	KTS Carrier	gnomeAD_EAS	ACMG Classification
chr16	84189298	DNAAF1	NM_178452:exon5:c. 685C > T:p.H229Y	KT2, LEI	0.0198	Uncertain significance
chr14	50092598	DNAAF2	NM_001083908:exon2:c. 2032A > G:p.T678A	KT2	0.0185	Likely benign
chr3	52396410	DNAH1	NM_015512:exon31:c. 4987C > T:p.R1663C	KT2	0.0129	Likely benign
chr17	78061565	CCDC40	NM_017950:exon15:c.2609G > A:p.R870H	LEI	NA	Uncertain significance
chr17	72308221	DNAI2	NM_001172810:exon12:c.1538C > T:p.A513V	KT1	0.0093	Likely benign
chrX	38146295	RPGR	NM_001034853:exon15:c.1957G > A:p.G653S	KT1	NA	Likely benign
chr3	52426507	DNAH1	NM_015512:exon64:c.10080G > C:p.Q3360H	KT4	0.0136	Uncertain significance
chr17	78024067	CCDC40	NM_017950:exon7:c.1144G > A:p.E382K	KT5	0.0099	Likely benign
chr7	151797901	GALNT11	NM_001304514:exon2:c.71G > A:p.R24H	LEI,KT4,KT5	0.0426	Uncertain significance

**Table 3:** Variants pathogenicity assessment and interpretation by the ACMG guideline.

Chr	Start	refGene	AAChange	KTS Carrier	gnomeAD_EAS	Segregation		ACMG Classification	Causative
						Paternal	Maternal		
chr3	52429325	DNAH1	NM_015512:exon69:c.10970C > G:p.T3657R	KT2	0.0037	Yes	Unavailable	Uncertain significance	Compound heterozygous
chr7	21641058	DNAH11	NM_001277115:exon18:c.3470T > G:p.L1157R	KT2	0.0025	Yes	Unavailable	Uncertain significance	
chr17	78071197	CCDC40	NM_017950:exon19:c.3175C > T:p.R1059X	LEI	0.0001	No	Yes	Pathogenic	Stopgain
chr3	52360191	DNAH1	NM_015512:exon4:c.442C > T:p.R148C	KT1	NA	Yes	Unavailable	Uncertain significance	Compound heterozygous
chr3	52387194	DNAH1	NM_015512:exon19:c.3103C > T:p.R1035C	KT1	0.0012	No	Unavailable	Uncertain significance	Compound heterozygous
chr9	34501139	DNAI1	NM_001281428:exon12:c.1035T > G:p.N345K	KT1	NA	Yes	Unavailable	Uncertain significance	
chr8	133673819	LRRC6	NM_001321961:exon2:c.64dupT:p.S22fs	KT1	NA	No	Unavailable	Pathogenic	Homozygote
chr5	13789010	DNAH5	NM_001369:exon51:c.8462T > C:p.L2821P	KT4	NA	No	Yes	Uncertain significance	Compound heterozygous
chr5	13830706	DNAH5	NM_001369:exon36:c.6061G > C:p.G2021R	KT4	NA	Yes	No	Likely pathogenic	Compound heterozygous
chr3	52380653	DNAH1	NM_015512:exon11:c.1822C > T:p.Q608X	KT5	NA	Unavailable	Yes	Pathogenic	Stopgain

#### Variants Validation and Pathogenicity Assessment

**Figure 3:**
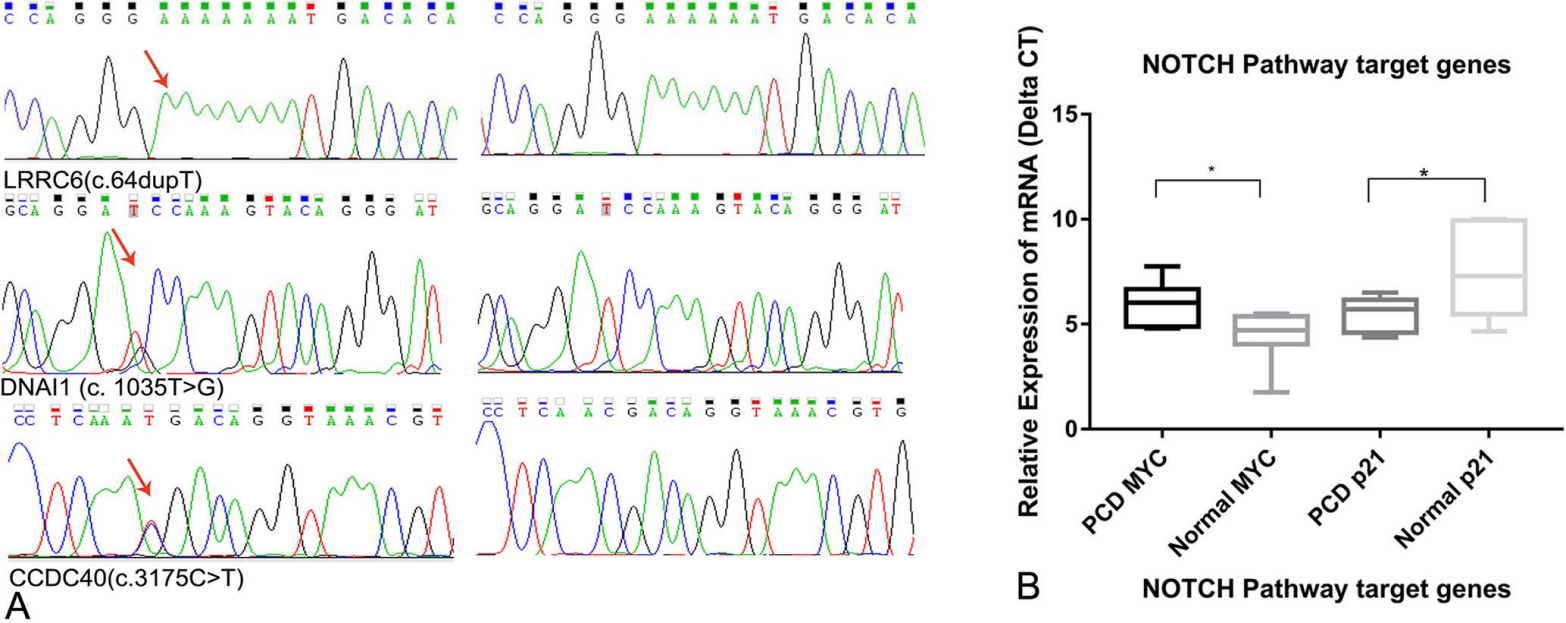
**(A)** Sanger validation results of the variants *DNAI1* (c. 1035T > G), *CCDC40* (c. 3175 *C* > T), and *LRRC6* (c.64dupT). **(B)** Relative expression of the NOTCH pathway target genes *MYC* and *p21* (*p<0.05).

ACMG prediction and functional analysis showed that four variants are pathogenic, and six variants are of uncertain significance, which were considered KTS-associated variants (**Table 3**). Sanger sequencing validated all candidate variants including *LRRC6*, *DNAI1*, and *CCDC40* (**Figure 3A**). Our study also showed the two rare variants in *DNAH5* of KT4 were inherited from the mother and father, respectively, indicating that they are compound heterozygous mutations (**Table 3**, **Supplemental Figure S1**). The variant of c.6061G > C in *DNAH5* changes the last nucleotide of exon 36 and completely abolishes the splicing signal, which indicated that it is more likely a pathogenic variant than a VUS. Only c.10970C > G of *DNAH1* was presented in the father of KT2, indicating that c.4987C > T and c.10970C > G are also highly likely to be compound heterozygous mutations. The variants in *DNAH11* might contribute to the phenotype of KT2. Hence, these identified compound heterozygous mutations were the genetic factors, which might cause the KT1, KT2, and KT4 phenotype disorder, but more evidence is needed.

The two heterozygous pathogenic variants were present in LEI and KT5, but this alone does not explain KTS in LEI and KT5, respectively. Since KTS is a recessive genetic disorder, the phenotypic defect in LEI and KT5 may be caused by trans-heterozygous interactions between the carried causative gene and other PCD genes (Li et al., 2016). In summary, in our study, 10 KTS-associated variants were identified among six causative genes of KTS, including four pathogenic variants.

#### Activation of the NOTCH Signaling Pathway by GALNT11

Real-time expression analysis showed that the expression of *MYC* and *p21* was significantly different among patients with KTS and normal individuals. The expression of *p21* was up-regulated by 5.03-fold in patients with KTS compared with that in normal individuals, but that of *MYC* was down-regulated by 0.29-fold (**Figure 3B**). Our study demonstrated the NOTCH1 pathway might be abnormally activated in patients with KTS, but the underlying mechanism is still unknown. No significant difference was found in terms of NOTCH1 pathway target gene expression among GALNT11 mutation carriers and controls.

### Discussion

In this study, we investigated five Chinese patients with KTS and identified 10 KTS-associated variants that overlapped in six causative genes, namely, *CCDC40*, *DNAH1*, *DNAH5*, *DNAH11*, *DNAI1*, and *LRRC6*. Compound heterozygous mutations were found in *DNAH1* and *DNAH5*. Further analyses showed that the NOTCH pathway might be activated in patients with KTS. Our study showed that patients with KTS carried multiple PCD candidate genes with rare compound heterozygous mutations in causative genes of *DNAH1* and *DNAH5*.

Primary ciliary dyskinesia is remarkably heterogeneous although its inheritance mostly follows an autosomal recessive pattern (Djakow et al., 2016). To date, various gene mutations in over 40 causative genes have been reported, and approximately 35% of PCD remain genetically elusive (Kurkowiak et al., 2015; Yang et al., 2018). A series of monogenic mutations has been reported to contribute to the abnormal structure of motile cilia. The complete or partial deletion of IDAs or ODAs is a common abnormality. Ten KTS-associated variants in six causative genes were identified in our Chinese patients with KST. Defects in the *DNAH5* gene could cause ODA deletion (Hornef et al., 2006). Two novel heterozygous mutations in the *DNAH5* gene were observed in one patient with PCD, which might be highly deleterious, using *in silico* prediction methods. One stop–gain mutation was found in *CCDC40*. Pathogenic mutations in *CCDC40* cause IDA defects and microtubular disorganization (Becker-Heck et al., 2011). The frame-shift homozygous mutation of *LRRC6* might cause both ODA and IDA defects (Kott et al., 2012). The present study showed that most high-frequency mutations were present in the causative gene *DNAH1*. Six mutations in *DNAH1* were identified in all individuals, indicating that *DNAH1* might be a high-risk causative gene in Chinese patients with KTS. The other mutations were identified in PCD causative genes, namely, *DNAI2*, *DNAAF1*, *DNAAF2*, and *RPGR*, but with uncertain significance (**Supplementary Table S3**). Further functional investigation would aid in confirming the role of these mutations in the motile cilia structure of KTS.

GALNT11 is a polypeptide N-acetylgalactosaminyl transferase that can catalyze the initiation of protein O-linked glycosylation. O-Glycosylation of NOTCH1 promotes its activation, modulating the essential balance between motile and immotile cilia in the left–right organizer to determine laterality (Boskovski et al., 2013). In the present study, we found a mutation of *GALNT11* in three patients with KTS. The missense mutation (c.G71A) was located in the highly conserved domain of *GALNT11*, and it might disturb the protein function. Therefore, we further analyzed the activation of the NOTCH pathway between patients with KTS and normal controls. Our results showed that the expression of down-stream target genes of *p21* was significantly up-regulated in patients with KTS. *GALNT11* might contribute to the phenotype of situs inversus in KTS.

We identified compound heterozygous mutations in the causative genes *DNAH1* and *DNAH5*. We only identified one homozygous mutation in the known candidate gene *LRRC6* in one patient with KTS. Our study showed that most of the known candidate genes present heterozygous mutations, but each patient with KTS carried multiple gene mutations. One limitation of this study is that DNA samples were not available for all the parents of the recruited KTS patients to confirm the inheritance mode of these mutations. It has been shown that compound heterozygous mutations can also cause extreme phenotypic diversity in the cilia ultrastructure of patients with PCD (Yang et al., 2018). It has also been shown that trans-heterozygous interactions between *DNAH6* and other PCD genes can potentially cause heterotaxy and ciliary dysfunction (Li et al., 2016). This indicates that heterotaxy might have a non-monogenic etiology. In summary, KTS is a complex genetic heterogeneity disorder that might be mediated by heterozygous mutations in multiple PCD- or cilia-related genes.

In conclusion, we identified 10 KTS-associated variants in six causative genes by targeted next-generation sequencing, including compound heterozygous mutations in *DNAH1* and *DNAH5*. The potential pathogenic gene *GALNT11* activated the NOTCH pathway, which might cause situs inversus in KTS. The various pathogenic mutations in different genes involved in KTS indicate genetic diversity and heterogeneity among patients with KTS. The identification of genetic defects that cause KTS is of great importance for its clinical diagnosis and genetic counseling.

## Data Availability

The raw data supporting the conclusions of this manuscript will be made available by the authors, without undue reservation, to any qualified researcher.

## Ethics Statement

The project was approved by the ethics committee of the Ethics Committee of Shenzhen People’s Hospital. Written informed consent was obtained from the patients for the publication of the patient’s identifiable information. All procedures performed in studies involving human participants in accordance with the 1964 Declaration of Helsinki ethical standards.

## Author Contributions

YY, YF, and BL prepared the project proposal and study design. YY and PZ conducted bioinformatics and statistical analysis of sequencing data. QH, PanZ, and ShuL conducted sample collection and Sanger sequencing validation. XT, ShengL, LC, and YF conducted clinical diagnosis of Kartagener syndrome. XH and LC assisted with the prepared and revised manuscript. All the authors have read and approved the final manuscript.

## Funding

The study was supported by the Guangdong Provincial Natural Science Foundation (2018A030310674), National Key Research and Development Program of China (2016YFC1304400), the Guangdong Provincial Science and Technology Project (2017A020214016), and Shenzhen Science and Technology Project (JCYJ20170413093032806, JCYJ20180305164128430).

## Conflict of Interest Statement

The authors declare that the research was conducted in the absence of any commercial or financial relationships that could be construed as a potential conflict of interest.

## Abbreviations

PCD, primary ciliary dyskinesia; KTS, kartagener syndrome; ODA, outer dynein arm; IDA, inner dynein arm; TEM, transmission electron microscopy; ACMG, American College of Medical Genetics and Genomics; VUS, uncertain significance variants

## Supplementary Material

Supplemental Figure S1 Sanger validation results of the compound heterozygous variants c.8462 > C and c.6061 > C in *DNAH5* of KT4
