# Supplementary figures and images for: Identification of Pathogenic Mutations and Investigation of the NOTCH Pathway Activation in Kartagener Syndrome
Source: Front Genet. 2019 Aug 22;10:749. doi: 10.3389/fgene.2019.00749 (PMC6713718; doi:10.3389/fgene.2019.00749)

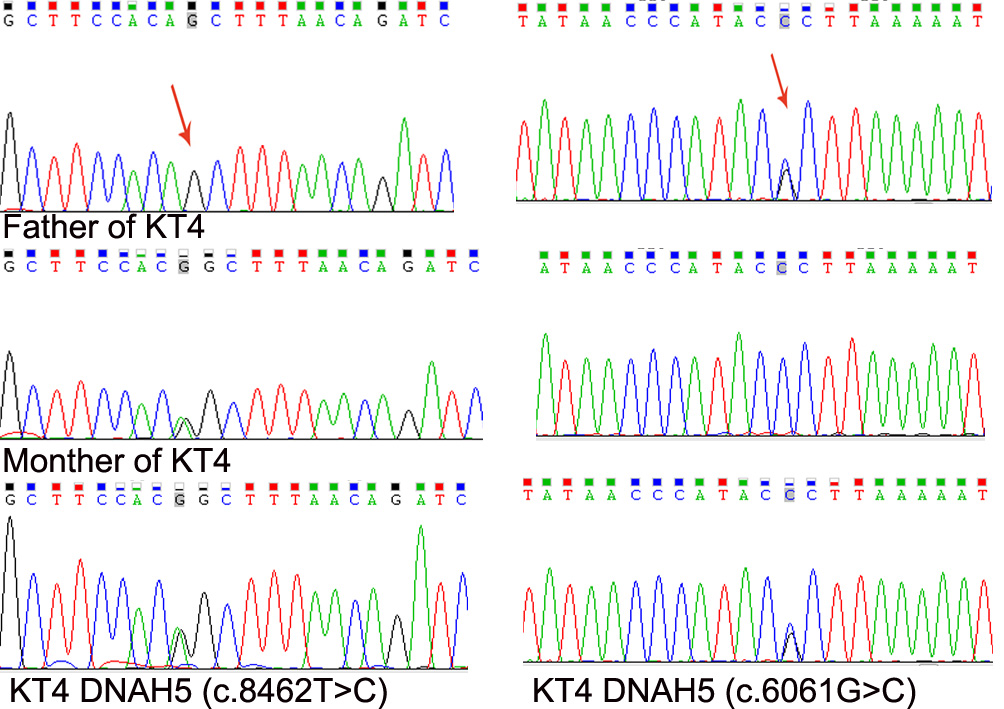

Supplement: Supplemental Figure S1 — Sanger validation results of the compound heterozygous variants c.8462 > C and c.6061 > C in DNAH5 of KT4 [file Image_1.jpeg]
